# Le refus des malades en réanimation: enquête auprès de 100 médecins réanimateurs marocains

**DOI:** 10.11604/pamj.2020.37.239.17651

**Published:** 2020-11-16

**Authors:** Boubakar Charra, Amine Raja

**Affiliations:** 1 Département d’Anesthésie et de Réanimation Centre Hospitalier Universitaire Ibn Rochd, Faculté de Médecine et de Pharmacie, Université Hassan II Casablanca, Casablanca, Maroc

**Keywords:** Réanimation admission, refus, triage

## Aux éditeurs de Pan African Medical Journal

Les services de soins intensifs sont des lieux dans lesquels sont accueillis des patients souffrant des pathologies potentiellement graves, et où les soins reposent sur des techniques de support artificiel. La mission du réanimateur est d´accepter l´admission d´un patient en réanimation. Néanmoins, pour des raisons variables, celui-ci peut être amené parfois à refuser l´admission d´un patient qui lui est proposé. La fréquence des refus d´admission et les facteurs menant les réanimateurs à prendre cette décision varient d´une étude à l´autre [1]. Les objectifs de notre travail sont de contribuer à apporter une meilleure connaissance du processus d´admission des malades en réanimation dans nos hôpitaux, avoir une idée sur l´avis des médecins réanimateurs sur le triage et le refus des malades en réanimation, ainsi que les facteurs menant à prendre ces décisions.

Enquête par sondage menée auprès d´un groupe composé de 100 médecins réanimateurs marocains durant un an. En profitant des congrès organisés au cours de cette période pour distribuer les questionnaires aux médecins. La méthodologie choisie a été celle d´un questionnaire anonyme composé de questions ouvertes, semi-ouvertes et fermées.

L´échantillon comporte 100 médecins dont 58% sont des hommes et 42% sont des femmes, dont l´âge moyen des médecins interrogés est de 33,76 ans, ont un exercice en réanimation depuis une durée moyenne de 6,11 ans, avec des extrêmes de 2 mois et 25 ans. Quarante pourcent des médecins interrogés affirment l´existence d´une réflexion quant à l´admission des malades dans les services où ils exercent. Tandis que 57% des médecins interrogés affirment l´inexistence d´une réflexion collégiale quant à l´admission des malades dans les services où ils exercent. Les grandes lignes de cette réflexion varient d´un médecin à l´autre. La plupart d´entre eux ont déclaré qu´ils admettent les malades souffrant de détresses vitales en prenant en considération la capacité litière du service seule (26,6% des réponses) Tableau 1. Quatre-vingt-trois pourcent des médecins interrogés ont répondu spontanément à la question concernant les facteurs menant le plus souvent au refus des malades en réanimation, sans avoir à choisir parmi une liste d´éléments proposés. Globalement, on peut classer les facteurs de refus cités dans les réponses des médecins en quatre groupes: 1) des facteurs liés au service; 2) des facteurs liés à la pathologie du patient; 3) des facteurs liés au malade lui-même ; 4) des facteurs liés à la structure hospitalière.

**Tableau 1:** les grandes lignes, et nombre (n) de médecins l´ayant cité

Les grandes lignes	n	Pourcentage(%)
Admettre les malades souffrant de détresses vitales en prenant en considération la capacité litière du service, seule	8	26,6
Admettre les malades dont le type de leurs pathologies relève du service (médicale ou chirurgicale)	3	10
Discuter les cas difficiles au cours des staffs avant de prendre la décision de les admettre au service	3	10
Donner la priorité d´admission aux sujets jeunes	3	10
Donner la priorité d’admission aux sujets «qu´on peut sauver»	3	10
Eviter l´admission des sujets âgés moribonds	1	3,3
Admettre les malades ayant des pathologies «curables»	1	3,3

Le facteur le plus souvent cité dans les réponses des médecins est le manque de place au niveau des services de réanimation (49,4% des réponses), suivi de la stabilité de l´état clinique (15,6% des réponses) et le mauvais pronostic des malades (15,6% des réponses) à l´admission, alors que l´âge avancé des malades arrive en troisième lieu (13,2% des réponses). Parfois l´absence de certaines spécialités, et l´impossibilité de réaliser des examens complémentaires ainsi que des gestes thérapeutiques dans le même hôpital (8,4% des réponses), l´un des facteurs menant le plus souvent au refus des malades proposés pour une admission en réanimation (Tableau 2). Cinquante-quatre pourcent des médecins déclarent être gênés dans leur exercice par une inadéquation de leurs capacités de soins par rapport aux demandes qui leur sont faites, dont 72,2% (39 médecins) parmi eux ont expliqué les répercussions de cette situation dans leur pratique quotidienne. Par contre 43% des médecins ne déclarent pas être gênés dans leur exercice par une inadéquation de leurs capacités de soins par rapport aux demandes qui leur sont faites. Les répercussions de cette situation sont nombreuses selon les réponses des médecins interrogés, on les a classé en trois groupes (Tableau 3): des répercussions psychologiques sur les médecins a constitué l´élément le plus présent dans les réponses des médecins à cette question (46,1% des réponses), des répercussions négatives sur la qualité des soins offerts (35,9% des réponses), des répercussions sur le malade. Cinquante-deux pourcent des médecins sont d´accord pour l´adoption d´une politique de triage des malades proposés pour une admission en réanimation. Les critères de triage cités par les médecins interrogés sont (par ordre de fréquence décroissant dans les réponses des médecins): le degré de gravité de la pathologie (36,3% des réponses), l´âge du malade (29,5% des réponses), son pronostic (20,4% des réponses), la réversibilité de sa pathologie (6,8% des réponses ), l´état clinique du malade à l´admission (4,5% des réponses), et le terrain sous-jacent (2,2% des réponses) (Tableau 4). La majorité des médecins interrogés (85%) souhaite l´ouverture d´une discussion sur l´admission des malades en réanimation afin d´établir une politique d´admission commune.

**Tableau 2:** les facteurs menant au refus des malades en réanimation, et nombre (n) de médecins l´ayant cité

Facteurs de refus	n	Pourcentage %
**Les facteurs de refus liés au service**		
Le manque de places	41	49,4
Le manque de ressources humaines médicales et paramédicales	4	4,8
Le manque de moyens de monitorage	3	3,6
**Les facteurs de refus liés à la pathologie**		
Pathologie de mauvais pronostic	13	15,6
Pathologie grave	8	9,6
Pathologie incurable	7	8,4
Bilan diagnostique incomplet	2	2,4
Pathologie qui ne relève pas du service (malade ayant une pathologie médicale proposé à un service de réanimation chirurgicale par exemple)	2	2,4
Mort cérébrale	1	1,2
**Les facteurs de refus liés au malade**		
Malade en état clinique stable (ne nécessitant pas un milieu de réanimation	13	15,6
L´âge avancé	11	13,2
Présence de tares	2	2,4
**Les facteurs de refus liés à la structure hospitalière**		
L´absence de certaines spécialités	3	3,6
L´absence de certains examens complémentaires	2	2,4
Le manque de moyens thérapeutiques (unité de dialyse par exemple)	2	2,4

**Tableau 3:** inadéquation des capacités de soins par rapport à la demande et ses répercussions

Répercussions	n	Pourcentage %
**Répercussions psychologiques**		
Le sentiment d´épuisement	5	12,8
Le sentiment d´insatisfaction	3	7,7
La déception	2	5,1
L´inéfficacité	2	5,1
Le stress	2	5,1
L´irritabilité	1	2,5
Le surmenage	1	2,5
Le sentiment de frustration	1	2,5
Les conflits entre collègues	1	2,5
**Répercussions sur la qualité des soins**		
Diminution de la qualité des soins offerts et mauvaise prise en charge des malades	11	28,2
Perte de temps et retard de prise de charge des malades	3	7,7
**Répercussions sur le malade**		
Augmentation du taux de mortalité	4	10,2
Aggravation du pronostic	2	5,1
Manque de place pour les malades nécessitant une hospitalisation	2	5,1

**Tableau 4:** les critères de triage des malades proposés pour une admission en réanimation, nombre (n) de médecins l´ayant cité

Critères de triage	n	Pourcentage %
Le degré de gravité de la pathologie	16	36,3
L´âge du malade	13	29,5
Le pronostic du malade	9	20,4
La réversibilité de la pathologie	3	6,8
L´état clinique du malade à l´admission	2	4,5
Le terrain sous-jacent (présence de tares)	1	2,2

Malgré le fait qu´il constitue un sujet de discussion polémique, le refus des malades en réanimation existe bel et bien et sa fréquence varie d´un service à l´autre [1], il concerne entre 10 et 35% des malades présentés aux services de réanimation selon des études publiés à ce sujet [1,2]. Beaucoup de travaux ont été réalisés afin d´étudier les facteurs associés au refus des malades en réanimation d´une façon globale. Alors que certains auteurs ont étudié ces facteurs selon le type de pathologie, en répartissant les malades en groupe dont la nature de la pathologie constitue une caractéristique commune [2-5]. L´étude de Garrouste Orgeas [6] a identifié les facteurs indépendants associés à un refus d´admission en réanimation. Les patients non admis en réanimation étaient plus souvent aidés à domicile ou complètement dépendants, avaient plus de cancer avec métastases prouvées et quand le service de réanimation manquait de place. Dans une étude d´Azoulay [7] les facteurs associés aux décisions de non admission étaient l´âge > à 65ans, les diagnostics suivants à l´admission: cancer métastasé sans espoir de rémission… En revanche dans la même étude, les récidives de leucémies avec défaillance multiviscérale ou insuffisance respiratoire aigüe et les comas végétatifs n´étaient pas associés à un refus d´admission. Dans l´étude de Dumont R *et al*. [8], 382 patients ont été refusés dont 31 parmi eux ont été transférés pour prise en charge spécialisée dans un autre centre hospitalier, et 7 non admis par refus des proches ou du patient. Les 344 patients non admis ont été répartis en 3 groupes à savoir Groupe A: patients refusés parce qu´ils étaient dans un état jugé « trop grave », B: patients refusés parce qu´ils était dans un état jugé « pas assez grave », C: patients refusés « faute de place ». Le principal motif de refus dans cette étude a été un état jugé « pas assez grave ». Et Le principal motif de refus des patients du groupe A (état trop grave), était un mauvais pronostic vital à court terme (35,6%), une perte d´autonomie préexistante (28,9%) et une pathologie chronique sévère (22,2%). Aucun patient proposé pour traumatisme ou tentative de suicide n´a été refusé pour un état jugé « trop grave ». Cette étude s´est attachée a identifié les facteurs associés à un refus d´admission pour les patients présentant un état clinique trop grave. Ces patients étaient significativement plus vieux, plus porteurs de pathologies chroniques antérieures, moins autonomes et présentaient plus de défaillances vitales au moment de la proposition d´admission [8].

Dans l´étude René Robert *et al*. [9]. 1 762 patients, ont été refusé, dont 116 avec une admission précédemment refusée dans une autre unité de soins intensifs (USI) et 270 parce qu'ils étaient jugés trop malades ou trop bien pour bénéficier de l'admission en USI. Sur les 1 332 patients restants, 1 139 ont été admis et 193 ont été refusés par manque de place. Dans l’étude de Garrouste Orgeas *et al*. [10], 55,4% des malades ont été refusés parce qu´ils étaient dans un bon état, 37,4% pour un état très grave, 6,5% pour manque de place, et un malade pour refus d´hospitalisation par sa famille. Dans l´étude de MohammediI *et al*. [11] réalisée sur 251 patients proposés, les causes de non admission étaient le manque de place dans 92% des cas, la gravité excessive de l´état du malade à l´admission dans 4% des cas, et le manque de gravité de l´état du malade à l´admission dans 4% des cas. Parmi les facteurs de non admission déjà cités dans les études, on remarque que le nombre de place disponible et l´âge avancé des malades proposés pour une admission en réanimation sont fréquemment observés. En effet, ces deux facteurs ont fait l´objet de beaucoup d´études, on va rapporter dans ce travail une partie de ces études: 1) Vincent JL [12] a mené une enquête sur 504 réanimateurs de tous les pays d´Europe de l´ouest, par la société européenne de soins intensifs, qui constate que le nombre de lit en réanimation représente souvent un obstacle à l´admission de malades graves [2]. 2) Dans l’étude de Metclave *et al*. [13] le refus des malades à cause d´un manque de lits a été observé dans 56% des cas. Dans l´étude de Dumont R *et al*. [8] le refus à cause d´un manque de place a été observé dans 13,9% des cas.

Dans notre étude, le manque de lits occupe la première place parmi les facteurs de refus des malades en réanimation cités par les médecins interrogés (49,4% des réponses à la question concernant les facteurs de refus des malades en réanimation). L´influence de l´âge sur l´admission des malades en réanimation est une question intéressante qui a suscité l´intérêt des réanimateurs: a) dans l´étude de Chelluri *et al*. [14], la survie des patients de plus de 85 ans hospitalisés en réanimation est de 65%, chiffre inférieur aux données de l´ensemble de la population. B) Dans l´étude Guidet B *et al*. le risque de décès des patients admis augmente de 1% par année d´âge de 18 à 70 ans, et de 2% par année d´âge pour les patients de plus de 70 ans [15]. Dans une autre étude de María-Consuelo *et al*. [16], 338 patients âgés ont été évalués pour l´admission en USI et 88 ont été refusés (26%). Les patients refusés parce qu'ils étaient «trop malades pour en bénéficier» présentaient plus de comorbidité et une situation fonctionnelle et mentale plus grave, avec une mortalité à un an également plus élevée 73,7%. C) Cohen [17] s´intéressant, à partir d´une importante base statistique, au devenir du patient ventilé mécaniquement en fonction de l´âge, 70% des patients de plus de 85 ans décèdent à l´hôpital contre 32% des patients de moins de 29 ans. Dans notre étude l´âge avancé des malades proposés pour une admission en réanimation arrive en troisième lieu parmi les facteurs de refus cités par les médecins interrogés (15,6% des réponses à la question concernant les facteurs de refus des malades en réanimation). Concernant le devenir des patients non admis en réanimation, il n´existe que peu d´études montrant le devenir des patients après un refus dans un service de réanimation. La première étude réalisée sur le sujet trouve qu´à niveau de gravité similaire, la mortalité des patients admis est identique à la mortalité prédite, alors qu´elle est multipliée par 2,5 par rapport à la mortalité prédite chez les patients refusés [18]. Une étude cohorte britannique confirme ces données en montrant que les patients refusés ont un risque relatif de décès de 1,6 par rapport à ceux admis en réanimation [13].

## Conclusion

Le refus d´admission des malades en réanimation est une question d´actualité, fort intéressante. Le taux de refus des malades proposés pour une admission en réanimation varie considérablement d´un service à l´autre. Les causes de refus sont multiples. Concernant le devenir des patients refusés, les études ont montrés qu´en comparaison aux patients admis, la mortalité est globalement plus importante pour les malades non admis. La Society of Critical Care Medicine (SCCM) et quelques auteurs ont proposé des recommandations concernant l´admission des malades en réanimation, dont l´objectif essentiel est la gestion rigoureuse et efficace des soins, dans un service où les soins prodigués relèvent d´une médecine très technique et onéreuse, pratiquée par un personnel hautement qualifié. Ces recommandations sont itératives dans le temps, afin de suivre le progrès de la réanimation. Mais, elles sont difficilement applicables par les médecins pour différentes raisons. L´éthique, plus qu´une théorie, est d´abord une pratique médicale.
